# Primary Malignant Melanoma of the Genitourinary System: A Systemic Review and Report of Eight Cases

**DOI:** 10.7759/cureus.30444

**Published:** 2022-10-18

**Authors:** Azadeh Khayyat, Mohammad Ali Esmaeil Pour, Seyedreza Mousavi, Amir-Reza Khalili-Toosi, Ali Amin

**Affiliations:** 1 Pathology, Medical College of Wisconsin Affiliated Hospitals, Milwaukee, USA; 2 Internal Medicine, University of North Carolina (UNC) Health Blue Ridge Hospital, Morganton, USA; 3 Pathology, Beth Israel Deaconess Medical Center, Harvard Medical School, Boston, USA; 4 Pathology and Laboratory Medicine, St. Paul Hospital, Providence Healthcare, Vancouver, CAN; 5 Pathology and Laboratory Medicine, Brown University, Providence, USA

**Keywords:** radiotherapy, methotrexate, immune check point inhibitors, ctla-4 inhibitors, pd-1 inhibitors, immunotherapy, chemotherapy, vascular endothelial growth factor, genitourinary malignant melanoma, mucosal malignant melanoma

## Abstract

Malignant melanoma (MM) of mucosal membranes (excluding anus and head-neck) is a rare but aggressive disease with poor outcomes. The knowledge of this tumor's development, etiology, and management is scarce, mainly due to the low case numbers. We presented eight cases and performed a comprehensive literature review on mucosal MM (between 1970 and 2020). We identified 47 manuscripts on 55 patients with primary mucosal MM (limited to urothelium and vagina) and reviewed demographics, tumor specifications (morphology, stage, etc.), management, and survival. We identified 10 manuscripts discussing treatment in 1595 mucosal and non-mucosal MM and extracted the data regarding the non-surgical treatment modalities of mucosal MM patients.

In 63 cases, 48 tumors primarily occurred in the urothelium (urethra: 47, bladder: 1) and 15 in the vagina. Molecular studies in a subset of cases revealed alterations in c-KIT, NRAS, BRAF (non-V600E and V600E), TP53, and NF1. Fifty-three patients underwent surgery (with additional chemotherapy, immunotherapy, and radiotherapy in 19, eight, and eight patients, respectively). The outcome was available in 52 cases, showing 21 deaths, 10 without recurrence, two alive with disease, and five lost to follow-up. Shared genetic signatures in mucosal and skin MM suggest a similar development mechanism; however, unlike skin MM, there are less BRAF mutations and more PI3K/AKT/mTOR pathway alterations in mucosal MM. Prolonged chemotherapy (i.e., methotrexate) and immune-modulating agents (i.e., natalizumab) may be risk factors. The stage at diagnosis and proper surgical extirpation are keys to the prognosis and survival of patients.

## Introduction and background

Malignant melanoma (MM) is a common cutaneous malignancy causing the highest skin cancer-related death in North America [1]. MM can rarely develop in mucosal tracts. Genitourinary malignant melanoma (GUMM) involving the female genital tract and urethra in both sexes is a sporadic type of melanoma that comprises less than 1% of all melanomas in women and less than 0.1-0.2% of all melanomas [2-9]. The annual incidence of urethral melanoma in the United States is around 1.5 per million women [6]. Because of its low incidence, this type of melanoma is not well studied. Some case studies report a very aggressive course with poor outcomes [10-12]. In this systematic review, we provide a comprehensive review of available literature on GUMM with a focus on the risk factors and genetic profile of this entity to shed light on the possible etiologies.

## Review

We conducted a systematic review of the literature. Our study's primary aim was to evaluate demographic characteristics, risk factors, molecular signatures, treatment, and survival data. The search was performed using PubMed between 1970 and 2020 using the following keywords: (((“melanoma” [MeSH terms] OR “melanoma” [all fields] OR (“malignant” [all fields] AND “melanoma” [all fields]) OR “malignant melanoma” [all fields]) AND (“urethra” [MeSH terms] OR “urethra” [all fields] OR “urethras” [all fields] OR “urethrae” [all fields]) AND 1970/01/01:2020/12/31 [date - publication]) OR ((“melanoma” [MeSH terms] OR “melanoma” [all fields] OR (“malignant” [all fields] AND “melanoma” [all fields]) OR “malignant melanoma” [all fields]) AND (“vagina” [MeSH terms] OR “vagina” [all fields] OR “vaginas” [all fields] OR “vagina s” [all fields] OR “vagina” [all fields]) AND 1970/01/01:2020/12/31[date - publication])) AND (1970:2020[pdat]).

Later, we limited our search to organs normally devoid of melanocytes. We excluded any hair-bearing epithelium (i.e., vulva, penis) for the final analysis. Also, anal melanoma and head-neck melanomas were excluded from this systematic review. We made every effort to avoid repeated cases. Our study included demographic factors age, gender, tumor location, signs and symptoms, type of treatment, risk factors and mutations, outcome, and recurrence. In addition, we identified ten papers discussing treatment in 1595 MM cases (mucosal and non-mucosal). We extracted the data regarding the non-surgical treatment modalities and outcomes of the mucosal MM patients. We also performed an extensive search in the archives of the Department of Pathology, Rhode Island Hospital, for the same time for mucosal MM cases and reviewed their available medical records.

Results

We identified 65 articles on GUMM published in the last 50 years, including case reports and case series. The demographics and disease details are provided in Table 1. In 63 cases, 48 lesions were located in the urothelium (urethra: 47, bladder: 1), followed by 15 in the vagina. We found eight in-house GUMM cases in the archives of the Department of Pathology, Rhode Island Hospital (RIH). None of the patients in this series had any other known primary source of MM. Overall, the cases of GUMM were more common in females (female: 42, male: 12). In urothelium alone, the F/M ratio was close to 3 (32/12). A complete pathological stage was provided in a few (n=6) cases (stage 1=1, stage 2=4, stage 4=1); however 23 patients had distant metastasis (liver, lung, brain, bone and pelvic, retroperitoneal and inguinal lymph nodes), compatible with stage 4 disease. Molecular studies in a subset of cases revealed alterations in c-KIT, NRAS, BRAF (non-V600E and V600E), TP53, and NF1 (Table 2). Molecular studies in RIH archival cases revealed a c-KIT mutation in one case of urethral MM and the absence of BRAF mutation in one anterior vaginal MM. Treatment data were available in 63 cases. Fifty-three patients were managed by surgery, followed by additional chemotherapy (19), immunotherapy (8), and radiotherapy (8). The remainder did not undergo surgery and received palliative care (chemotherapy only (3), radiotherapy only (2), chemo/radiation (1)). The outcome was available in 52 cases, showing 21 deaths, 10 patients without recurrence, and two patients alive with disease. Five patients were lost to follow-up. Table 3 provides the details of the literature on the non-surgical management of MM (including old and novel immunotherapeutic agents, chemotherapeutic agents, and radiotherapy).

**Table 1:** Demographics and disease details on mucosal MM. *c-KIT mutation present in the patient; received imatinib and pembrolizumab (nine cycles). **Patient received ipilimumab and nivolumab therapy. MM: malignant melanoma

Author/year	No.	Age/ sex	Site	Symptoms	Stage/size of the tumor	Treatment	Recurrence/outcome
Godec et al., 1981 [13]	1	54 F	Urethra	Palpable and visible mass in the distal urethra	Nodal (inguinal, groin, and suprapubic), small intestine, stomach, liver, and lung metastasis	Surgery	Dead of disease
McComiskeet al., 2015 [14]	1	76 F	Urethra	Vaginal pain, mass, and discharge	Size: 4 cm, T4N0M0	Surgery	No recurrence
Davuluri et al., 2019 [6]	1	73 F	Urethra	Vaginal pain and hematuria	Size: 4.9 cm; local invasion: vaginal mucosa; metastasis present	Surgery	Dead of disease
Aoki et al., 2019 [15]	1	77 M	Urethra	Mass, dysuria	Size: 6 cm pT3aN0M0 Stage IIA	Surgery	No recurrence
Safadi et al., 2017 [16]	1	52 F	Urethra	Spotting	Size: 3 cm	Surgery	-
Bhutani et al., 2017 [17]	1	70 F	Urethra	Mass, dysuria, obstruction, hematuria, blood spots	Size: 0.5 cm	Surgery	No recurrence
Sanchez-Ortiz et al., 2005 [18]	1	55 M	Urethra	Bleeding	1.0 mm depth T2 N0 M0	Surgery	No recurrence
Caputo et al., 2020 [19]	1	63 M	Urethra	Dysuria and blood spotting	Size: 1.5 cm metastasis (inguinal nodes and lung)	Surgery; immunotherapy	Recurrence (6 months)
Broussard et al., 2015 [20]	1	87 F	Urethra	Vaginal bleeding	Size: 5 cm	Surgery	Died (other causes)
Gupta et al., 2007 [21]	1	65 F	Urethra	Pallor, bleeding	Pagetoid spread in urothelium	Surgery	No recurrence
Sugaya et al., 1983 [22]	1	53 F	Urethra	Painless mass	Fingertip size metastasis present	Surgery	Dead of disease
Manivel and Fraley 1988 [23]	3	62 (Mean age) M	Urethra	(1) Bleeding, pigmented urethral meatus lesion; (2) metal blister and nodule; (3) mass in the fossa navicularis	(1) Size: 5 cm, thickness: 3.5 mm; (2) size: 2.1 cm, thickness: 5.5-6.0 mm; (3) size: 0.9 cm, thickness: 5 mm	(1) Surgery; (2) surgery, immunotherapy, radiotherapy; (3) surgery	(1) Lost to follow-up; (2) no recurrence; (3) lost to follow-up
Kim et al., 1993 [24]	1	59 F	Urethra	Mass	Size: 15 mm. Stage A	Surgery; chemotherapy (alpha-interferon)	No recurrence
Maestro et al., 2012 [25]	1	65 F	Urethra	Mass, obstruction	-	Surgery	No recurrence
Gassara et al., 2010 [26]	1	89 F	Urethra	Obstruction urgency dysuria hematuria	Size: 3 cm, local extension to bladder neck; metastasis (lung)	Chemotherapy	Dead of disease
Filipkowski et al., 2009 [27]	1	67 F	Urethra, bladder base	Organ prolapses, obstruction, dysuria, frequency	-	Surgery; chemotherapy	-
Sanders et al., 1986 [28]	1	71 M	Urethra	Nodule, obstructive symptoms	Size: 15 mm, depth: 7 mm invasion into corpus spongiosum 3 satellite lesions (largest 1.5 × 4 mm)	Surgery	Dead of disease
Das et al., 2021 [29]	1	40 F	Urethra	Vaginal swelling urinary incontinence	Size: 1 cm; metastasis present	Surgery and radiotherapy	Dead of disease
Begun et al., 1984 [30]	2	Mean age: 67 M	Urethra	(1) Bloody discharge, obstruction; (2) yellow then bloody discharge	(1) Metastasis present; (2) size: 2 cm, metastasis present	(1) Surgery; (2) surgery intravesical Bacillus Calmette-Guérin	(1) Dead of disease; (2) alive with disease
Chitale et al., 2001 [31]	1	64 M	Urethra	Hematuria, meatal ulcer, bleeding	Size: 1.5 cm	Surgery	No recurrence
Desgrandchamps et al., 1995 [32]	1	F	Urethra	-	-	Surgery	-
Mukai et al., 2003 [33]	1	72 F	Urethra	Hemorrhagic leukorrhea	Size: 2 cm, depth:5 mm; metastasis (lymph node)	Surgery; dacarbazine+ nimusutine+ vincristine+ interferon-β injection	Recurrence and metastasis
Kajikawa et al., 1987 [34]	1	76 F	Urethra	Anemia, jaundice, urethral mass, easily bleeding	Large mass, metastasis (retroperitoneal node)	Combined chemotherapy	Died (other causes)
Katz et al., 2005 [35]	1	74 M	Urethra	Hematuria, obstruction, urethral meatus mass	Size: 5.2 cm, Breslow index: 8.2mm	Surgery; chemotherapy	-
Takahashi et al., 1989 [36]	1	80 F	Urethra	urethral meatus mass, hematuria	Fingertip size mass	Surgery; chemotherapy	No recurrence
Yoshida et al., 1986 [37]	1	76 F	Urethra, bladder neck	Pain, dysuria	-	Surgery	No recurrence
Touyama et al., 1997 [38]	1	86 F	Urethra	Urethral bleeding	Size: 1cm, metastasis present	No surgery	No recurrence
Yoshizawa et al., 2007 [39]	1	69 F	Urethra	Mass	Size: 1 cm, T4N0M0 local invasion to bladder neck, vagina, and vulva	Surgery; immuno-chemotherapy	Dead of disease
Inoue et al., 2008 [40]	1	70 M	Urethra	Hematuria and urodynia	-	Surgery	Dead of disease
Arai et al., 1993 [41]	1	65 F	Urethra	4 months history of dysuria and 2 months history of urethral bleeding	Size: 2.5 cm, local invasion: vagina and bladder trigone	Surgery; adjuvant immuno-chemotherapy	Dead of disease
Nakamoto et al., 2007 [42]	1	75 F	Urethra	Hematuria and a small caruncle	Depth: 0.9 cm, stage II, metastasis to liver (in 3 months)	Surgery; dacarbazine+ interferon-β injection	No evidence of disease
Maruyama et al., 2014 [43]	1	94 F	Urethra	Dysuria and vulvar discomfort, hematuria	Size: 2 cm thickness ranged from 0.3 to 14 mm	Surgery	Dead of disease
Hansen et al., 2019 [44]	1	79 F	Urethra	Incontinence, dysuria two polypoidal protruded lesions	Size: 1.5cm, Clark level 4, Breslow index: 11mm T4b, N0, M0 stage IIc	Surgery	Dead of disease
Blaumeiser et al., 2000 [45]	1	66 F	Urethra	Local bleeding	-	Surgery	-
Pandey et al., 2014 [46]	1	62 F	Urethra	Obstruction, mass, bloody discharge	Size: 2 cm	Surgery	No recurrences
Methfessel et al., 1983 [47]	1	69 F	Urethra	-	-	Surgery; chemotherapy	-
Sanz Velez et al., 1989 [48]	1	74 F	Urethra	-	-	Surgery	Recurrence (5 years)
Günther et al., 2012 [49]	1	65 F	Urethra	Mass, bleeding	Size: 3 cm, thickness: 3.57 mm	Surgery	No recurrence
Thum et al., 1990 [50]	1	F	Urethra	-	-	Surgery	Dead of disease
Nakra et al., 2020 [51]	1	62 F	Urethra	Obstruction, pain, hematuria, weight loss, appetite loss	Size: 50 mm; metastasis present	Chemotherapy	Lost to follow-up
Richter and Lotze, 1985 [52]	1	38 F	Vagina	-	-	Chemotherapy; surgery	-
Betschart et al., 2007 [53]	1	F	Vagina	Protruding vaginal mass	Size: 6 cm, depth of invasion: 2.5 cm; metastasis present	Surgery; radiotherapy; chemotherapy	Recurrence (6 months)
Ghosh et al., 2007 [54]	1	60 F	Vagina	Protruding vaginal mass with bleeding	Size: 7 cm; high-risk Breslow’s system	Surgery; immunotherapy; chemotherapy; radiotherapy	Dead of disease (6 months)
Biswas et al., 2009 [55]	1	52 F	Vagina	Foul smelling bloody vaginal discharge, itching	Size: 4cm	Surgery; radiotherapy	-
Fukui et al., 2008 [56]	1	67 F	Vagina	Pain (sacralgia)	Metastasis to pelvis	Surgery; immuno-chemotherapy	No recurrence
Gökaslan et al., 2005 [57]	1	56 F	Vagina	Vaginal discharge, spotting	Size: 1.5-2 cm (depth of invasion > 2 mm), stage IV Clark classification	Surgery + immunotherapy	Dead of disease
Geisler et al., 1995 [58]	4	61.25 (mean age) F	3 in vagina 1 in urethra	Case (1) mass; case (2) spotting; case (3) mass; case (4) discharge	(1) Size: 8 mm; (2) size: 4.6 mm; (3) size: 12 mm; (4) size: 4 cm	Surgery (in all)	(1) No recurrence; (2) no recurrence; (3) no recurrence; (4) died (other causes)
Greggi et al., 2010 [59]	2	1) 43 F 2) 37 F	Vagina	(1) Hyperchromatic bleeding lesion	(1) Size: 6 cm, thickness: >20 mm stage II, American Joint Committee on Cancer stage IV A metastasis present; (2) size: 2 cm, thickness: >2mm	(1) Surgery, transdermal estrogen therapy, alfa-2B interferon therapy; (2) surgery	(1) Dead of disease; (2) dead of disease
Khayyat et al. (this study)	8	(1) 76 F; (2) 66 F; (3) 81 F; (4) 52 M; (5) 93 F; (6) 88 F; (7) 64 F; (8) 80 F	(1-3) Urethra; (4) bladder/ urethra; (5-8) vagina	(1) Bleeding; (2) bleeding, mass forming; (3) bleeding, mass forming; (5) bleeding; (7,8) bleeding	(1) Size: 0.8 cm, thickness: 8.5 mm, metastasis: bone, lung, adrenal (in 12 mo); (2) size: 1.5cm metastasis: lung; (3) size: 2.0cm, thickness: 0.6 cm, metastasis: liver (in 12 mo); (4) metastasis: liver; (5) metastasis: lymph node, brain; (6) metastasis: lung, liver, bone; (7) size: 4.0 cm, metastasis: liver, lung	(1) Surgery and radiotherapy; (2) surgery, immunotherapy^*^; (3, 4) surgery only; (5, 6) palliative radiotherapy (no surgery); (7) radiotherapy, chemo^**^; (8) N/A	(1) Dead of disease; (2) alive with disease; (3, 4) lost to follow-up; (5-7) dead of disease

**Table 2:** Frequency of molecular alterations in mucosal malignant melanoma.

Disease	Mutation				
	c-KIT	BRAF (V600E or non-V600E)	NRAS	TP53	NF1
Urothelium (urethra)	4-32%	14% (non-V600E)	13-21%	43%	14%
Vagina	25-44%	Rare	14%	25%	25%

**Table 3:** Literature on target therapy of malignant melanoma (including mucosal melanoma). *KIT mutation/amplification present. **Eight KIT mutations, 11 KIT amplifications, and five with both. ***Varied by KIT mutation status: 77% in mutated vs. 18% in amplified. ****Anti-PD-1 experienced better PFS than patients treated with anti-CTLA4. VEGF: vascular endothelial growth factor

Author/year	Case no./location	Stage	Immune checkpoint inhibitor anti-VEGF	Dacarbazine DAV-Feron carboplatin + paclitaxel, tyrosine kinase inhibitor	Outcome
Carvajal et al., 2011 [60]	28/acral, mucosal, and chronic sun damage	Mucosal melanoma with distant metastasis*	-	28 imatinib	Overall durable response rate: 16%; median time to progression: 12 weeks; median overall survival: 46.3 weeks
Robert et al., 2011 [61]	502	-	± ipilimumab	Dacarbazine	Ipilimumab + dacarbazine vs. dacarbazine (overall survival: 11.2 vs. 9.1 months) (survival rates at 1 year: 47.3% vs. 36.3%)
Hodi et al., 2013 [62]	24/mucosal melanoma, acral, chronic sun damage skin	Distant metastasis**	-	24 imatinib	Overall disease control rate: 50%***
D’Angelo et al., 2017 [63]	86 mucosal melanoma; 665 cutaneous melanoma	-	Nivolumab ± ipilimumab, 35 mucosal melanoma; 326 cutaneous melanoma	-	Nivolumab monotherapy, median progression-free survival: 3.0 mo. for mucosal melanoma and 6.2 mo. for cutaneous melanoma; nivolumab + ipilimumab, median progression-free survival: 5.9 mo. for mucosal melanoma and 11.7 mo. for cutaneous melanoma
Moya-Plana et al., 2019 [64]	Head and neck (18), vulvovaginal (12), acral (14)	Stage 3 (11), metastasis (11)	20 pembrolizumab, 24 ipilimumab	-	Median progression-free survival: ipilimumab 3 months, pembrolizumab 5 months
Indini et al., 2019 [65]	7 mucosal melanoma (vulva {2}, vagina {4}, cervix {1})	Metastasis (2 at Dx; 5 after Dx)	1 nivolumab, 2 pembrolizumab, 4 ipilimumab	-	Response rate to immune checkpoint inhibitor: 28.5%****
Sheng et al., 2019 [66]	29 mucosal melanoma	-	29 toripalimab, 29 axitinib	-	Median progression-free survival (in 14 cases): 7.5 months
Yan et al., 2021 [67]	114	Mucosal melanoma with distant metastasis	± bevacizumab	Carboplatin plus paclitaxel	Median progression-free survival - in carboplatin + paclitaxel+ bevacizumab arm: 4.8 months; in the carboplatin + paclitaxel arm: 3.0 months; median overall survival - in carboplatin + paclitaxel+ bevacizumab arm: 13.6 months; in carboplatin + paclitaxel arm: 9.0 months
Guo and Zhang, 2021 [68]	1 vagina	Stage 3 disease	Nivolumab	-	No recurrence (6 months)

Discussion

GUMM is a rare but aggressive disease with poor outcomes. There is no adequate information available on the disease and its course. Current knowledge suggests that GUMM's risk factors and pathophysiology differ from skin MM's [7]. A century ago, the first case of melanoma of the urethra was reported by Reed [69]. Since then, many cases have been reported. The urethral disease appears more prevalent in females, with a ratio of 3:1 [70]. Patients can be asymptomatic or manifest symptoms such as urinary obstruction, incontinence, dysuria, hematuria, vaginal bleeding, and melanuria. GUMM occurs in non-exposed mucosae; therefore, diagnosis is often late and requires clinical vigilance. Similar to the skin MM and because of the non-specific clinical features, the final diagnosis of GUMM is by histopathology. In the presence of detectable amounts of melanin pigment, microscopic diagnosis is not complicated; however, amelanotic and hypomelanotic melanomas may mimic other tumors like poorly differentiated urothelial or squamous cell carcinomas, sarcomatoid carcinoma, pleomorphic sarcoma, or extra-adrenal paraganglioma [71-74]. Unlike skin MM, GUMM can be amelanotic in up to 25% of cases [7].

Histologically, the neoplastic cells form nests, fascicles, storiform growth, or diffuse growth patterns. The tumor cells often reveal abundant eosinophilic cytoplasm with overtly pleomorphic nuclei showing coarse chromatin, multiple macronucleoli, and intranuclear inclusion. Intracytoplasmic melanin pigment may be present. Observation of the nested growth pattern in the tumor is a helpful diagnostic tool. However, fascicular, storiform and diffuse patterns are not uncommon, whereas rare patterns like pseudopapillary and pseudo glandular growth may cause confusion during a histological assessment [72,74]. Another helpful morphologic feature for recognizing primary melanoma is surface epithelial infiltration, either as a radial growth phase in which tumor cells infiltrate the basal layers or as the pagetoid spread to the full thickness of the epithelium. A search for this finding is encouraged to identify an in-situ component [72]. Ulceration may complicate the assessment of the in-situ component [75]. The tumor may show epithelioid and spindle neoplastic cells in pure or mixed populations. Other rare cell types observed in urothelial melanomas include rhabdoid, clear, small cell, and giant cell morphologies [72,75-77]. The tumor may rarely be mistaken for small cell carcinoma, lymphoma, and plasmacytoma. Careful assessment of morphology and administration of immunohistochemistry can be helpful in the differential diagnosis. Other diseases may clinically be considered in the differential diagnoses, including benign urethral lesions (including urethral mucosal prolapse, chancres, caruncle, and polyp), genital lentiginosis, and atypical melanocytic nevi, and atypical lentiginous hyperplasia [69,70]. Differential diagnosis by histomorphology is usually straightforward because these lesions do not share histomorphological features with GUMM, specifically having pleomorphic neoplastic cells.

Although rare, genitourinary melanoma may first present in urine cytology. Identification of melanoma cells in urine cytology, as well as melanuria or melanosis in the urine of patients with advanced metastatic melanoma, is an indication of the involvement of the genitourinary tract [71,72]. Therefore, awareness of cytomorphologic features of this entity should be considered to prevent misdiagnosis in urine cytology [73-76]. In urine cytology, melanotic-type GUMM exhibits large single cells or small clusters of malignant-appearing cells with intracytoplasmic melanin pigments. The cells contain enlarged eccentric nuclei, high nuclear to cytoplasmic ratio, and prominent nucleoli. Bi-nucleation, multi-nucleation, and occasional intranuclear inclusions are other observed features. Differential diagnoses of melanotic melanomas include pigments such as hemosiderin, lipofuscin, and melanosis [75-81]. Hemosiderin pigments presenting in histiocytes, and melanosis, defined as the deposition of melanin pigments in urothelial cells theoretically, are accompanied by benign nuclear features of their host cells. Lipofuscin pigments manifest in a similar fashion to the former pigments; however, they may rarely be observed in seminal vesicle cells presenting with atypical features similar to MM, but the perinuclear distribution of these pigments and the presence of sperm in the background can help distinguish them from MM [82-84]. The differential diagnoses of amelanotic GUMM may consist of high-grade urothelial carcinoma, prostatic adenocarcinoma, and other poorly differentiated malignancies from adjacent organs or distant metastases such as from the stomach or breast. In urothelial carcinoma, tumoral cells present with a high nuclear to cytoplasmic ratio. The enlarged nuclei are angulated, have irregular contours, and are markedly hyperchromatic with hardly visible nucleoli. Prostatic adenocarcinoma seldom is detected in urine cytology as cells with prominent nucleoli are usually arranged in cohesive clusters or acini. Lastly, metastatic tumors sometimes have specific features such as intracytoplasmic mucinous vacuoles in signet ring cell carcinoma of the stomach or linear arrangement of cells of metastatic lobular breast carcinoma that make them distinguishable from melanoma cells [75,76,82]. In these situations, the presence of accurate clinical information about other malignancies is vital. Once suspected, immunohistochemistry can play a significant role in the differential diagnosis.

Immunohistochemistry is a useful tool in differential diagnosis. Carcinomas and sarcomatoid carcinomas reveal strong expression of Cytokeratin and do not express melanocytic markers. A comprehensive panel including Cytokeratin, Vimentin, S100, SMA, and Desmin can help distinguish GUMM from pleomorphic sarcoma. Diffuse expression of S100 (in contrast to focal or negative expression in sarcomas with neuronal differentiation) is very helpful for this differential diagnosis. Other melanoma markers, including HMB45, SOX10, and Melan-A, are usually diffuse and strongly positive in GUMM, and cytokeratin is negative in all cases [3,85,86]. Paragangliomas can reveal focal S-100 expression in sustentacular cells compared to diffuse expression in GUMM. Neuronal and neuroendocrine markers like neuron-specific enolase (NSE) and synaptophysin highlight ganglion cells and are not expressed in GUMM. Differentiation of lymphoid neoplasms can be done using the specific lymphoid markers that are not expressed in melanoma. In the case of small cell carcinoma, expression of thyroid transcription factor 1 (TTF-1) (seen in up to 50% of small cell carcinomas) and proliferation index of >95% in the absence of melanoma markers can help in the differential diagnosis.

Normal mucosal membranes, including urothelium and vaginal mucosa, are usually devoid of melanocytes. Melanocytes that are the main tumoral cells in malignant melanoma embryologically originate from neural crest cells and are situated in the basal portion of the epidermis in the vagina in 3% of women. These cells can be aberrantly relocated in vaginal mucosa and can potentially cause melanoma in the vagina. GUMM can be seen anywhere in the mucosa; however, most common locations are close to some hair-bearing skin. The most common location of vaginal melanoma is in the anterior surface and lower half of the vagina, and they are mostly exophytic, polypoid, or pedunculated with secondary necrosis [86]. In the urinary tract, almost all MM are identified in the lower GU tract, and they are commonly seen in the distal portion of the urethra/vagina (Table 1). This finding supports the theory of local migration of melanocytes as the source of the MM. Metaplasia or misplacement of mesodermal and epithelial tissue is another proposed etiology. Bladder melanosis has also been reported as a risk factor for GUMM [84]. Neovaginal malignant melanoma was reported after operation and radiation therapy of vulvar squamous cell carcinoma (SCC) in a 71-year-old woman [85,86]. One study reported 20 patients diagnosed with vaginal malignant melanoma. All of them died with a median survival rate of 19 months [86]. The type B ultraviolet light (UVB) from sun exposure is the most important environmental risk factor in MM; however, it is not possible to blame deep-located GUMM on sun exposure. In addition, the belief that all mucosal melanomas are secondary has no merit. El-Safadi et al. reported that radiation therapy may be a predisposing factor in the development of melanoma in areas of the body without sun exposure [87].

While BRAF-V600E is the most common pathogenic mutation seen in cutaneous sun-exposed melanomas, mucosal and anogenital melanomas usually lack BRAF mutations and instead harbor KIT alterations. The American Joint Committee on Cancer staging guideline (American Joint Committee on Cancer eighth edition) recommends using cutaneous melanoma guidelines for vulvar melanoma staging and does not provide any recommendations for vaginal melanoma staging. In a study conducted by Zarei et al., they investigated the mutational status of invasive melanomas arising from different anatomic sites in the lower female genital tract (vulvar hair-bearing skin, glabrous skin, vagina, and urethra) in a group of 37 patients. Tumors were analyzed using a DNA-targeted next-generation sequencing panel covering the 21 most common genes and mutation hotspots in melanomas. The most common genetic alterations in invasive melanomas of the lower female genital tract are KIT (32%), TP53 (22%), and NF1 (19%). Pathogenic alteration in at least one of the MAPK pathway genes was shown in 66% (21/32) of cases. No statistical significance was seen between different primary tumor sites and the frequency of the oncogenic mutations, nor were any significant differences found by mutation status. Only one case of urethral melanoma showed a BRAF non-V600E mutation (D594G). Their results suggested similar molecular pathogenesis and overall survival in melanomas arising from the lower female genital tract, irrespective of their exact location in the urogenital area [86,87]. In another study performed by van Engen-van Grunsven et al., they evaluated a series of primary melanomas of the female urogenital tract for oncogenic mutations in KIT, NRAS, and BRAF in order to identify patients who may be amenable to targeted therapy. They reviewed 24 cases of female urogenital tract melanomas and used Sanger sequencing analysis for the detection of oncogenic mutations in exons 9, 11, 13, and 17 of KIT; exons 2 and 3 of NRAS; and exon 15 of BRAF. Twenty-four patients have included: fourteen vaginal melanomas, four cervical melanomas, five urethral melanomas, and one vulvar melanoma. NRAS mutations (4/24, 21%) were more prevalent than KIT mutations (1/24, 4%), while BRAF mutations were absent. Three of four NRAS mutations were present in vaginal melanomas (21%), mainly affecting codon 61 (3/4). They were mutually exclusive with the KIT mutation. The KIT mutation was present in a vaginal melanoma and affected exon 17. They concluded that the presence of NRAS mutation in melanomas of the female urogenital tract makes NRAS an interesting therapeutic target for these patients in the advanced setting. KIT mutations were rare in their study in contrast to other reports. They could not exclude that anatomical site-related differences and/or population-related differences in KIT mutation frequency exist within urogenital tract melanomas [88,89].

Some studies have identified an association between chemotherapy and the development of GUMM. Hansen et al. reported that a 79-year-old woman who received methotrexate for rheumatoid arthritis (RA) for 22 years was diagnosed with primary melanoma of the urethra [44]. Furthermore, Buchbinder et al. recently conducted a large cohort study in Australia and found that the risk of melanoma in RA patients on methotrexate is three times that of the normal population. A study by Polesie et al. revealed some association between melanoma and methotrexate use [89]. Other studies did not identify any plausible association between immunosuppressive therapy and the increasing incidence of melanoma [90,91]. Some authors have attributed this association to the strong association of RA with chronic inflammatory diseases [92]. Capkun et al. also reported a low incidence of melanoma in MS patients [91]. Sabol et al. assessed 145 patients and found an association between natalizumab with melanoma [92]. Seven cases with the diagnosis of MS on natalizumab were reported that were diagnosed with melanoma [91-96]. The median age was 41 years (range: 38-48 years). On the other hand, Nali et al. showed that by suppressing the expression of alpha-four integrin, which can inhibit melanoma metastasis, natalizumab could increase the chance of melanoma metastasis [96]. The association between immune checkpoint therapy and GUMM needs further clarification.

The treatment options are mostly limited to extensive surgery as these tumors are frequently found at high stages. In the early stages, radical surgery can control the tumor as initial therapy [3,97,98]. Almost 50% of patients show nodal involvement at diagnosis. Early diagnosis of the tumor and its recurrence can increase the survival rate [76,99] and poor outcome is usually the result of late diagnosis or misdiagnosis [76,99,100]. Vaginal melanoma and female urethral melanoma with a thickness of more than 3 mm benefit from being treated with pelvic exenteration. Surgery is ineffective if the tumor is aggressive or detected in higher stages. Chemotherapy is not a good choice in invasive tumors [97,98,101]. Vaginal melanoma has the ability to spread through blood, so early metastasis is seen more frequently, and the overall prognosis is poor. There are some poor prognostic factors such as age (>65 years), race (more in African American race), stage of the tumor, size (thickness >3.5 mm and diameter >15mm), ulceration, histologic type, lymph node involvement, presence of satellite lesion and metastasis (especially pulmonary) for this type of melanoma [5-9,12,72,77,102]. The presence of more than one mitotic figure, especially with a thickness of 1.5 mm, has been associated with a poor prognosis [3].

There is no specific guideline for chemotherapy in GUMM; the current regimens are based on the experience with cutaneous melanomas. However, in light of differences in the pathogenesis of cutaneous MM and mucosal MM, a difference in response to therapy is expected. The current guidelines for the treatment of cutaneous melanoma include [103]: (A) immunotherapy drugs (immune checkpoint inhibitors) such as pembrolizumab (Keytruda) or nivolumab (Opdivo) as the first drugs tried, especially in tumors without BRAF gene mutation. Ipilimumab (Yervoy), a different type of checkpoint inhibitor, is not typically used by itself as the first treatment, although it might be combined with nivolumab or pembrolizumab. (B) The cancer cells have BRAF gene mutation in about half of all melanomas. In these cases, targeted therapy with BRAF and MEK inhibitors is an option. Immune checkpoint inhibitors such as pembrolizumab or nivolumab are another option for these people. (C) A small portion of cutaneous melanomas has c-KIT gene alterations. These cases might benefit from targeted drugs such as imatinib (Gleevec) and nilotinib (Tasigna), although these drugs often stop working eventually. (D) Immunotherapy using interleukin-2 (IL-2) can help a small number of people with stage IV melanoma, and it might be tried if immune checkpoint inhibitors aren’t working. Higher doses of IL-2 seem to be more effective, but they can also have more severe side effects, so they need to be administered under close observation (in-patient).

Historically, dacarbazine-based chemotherapy was the mainstay of chemotherapy in advanced GUMM, mostly because of the absence of BRAF mutation in GUMM. Dacarbazine was formerly a conventional therapy for patients with previously untreated melanoma who did not have a BRAF mutation and, following dacarbazine therapy, had a median overall survival of 5.6-7.8 months [104]. In 2011, studies concluded that treatment with imatinib mesylate leads to substantial clinical responses in a subset of patients with advanced melanoma harboring KIT alterations and dacarbazine-based chemotherapy as first-line treatment in non-cutaneous metastatic melanoma: a multicenter, retrospective analysis in Asia [102]. Another study in the same year revealed that compared to dacarbazine + placebo, ipilimumab (at a dose of 10 mg/kg) in combination with dacarbazine enhanced overall survival in patients with previously untreated metastatic melanoma [102,104]. In 2013, Hodi et al. showed that amplification and mutations in the KIT proto-oncogene in subgroups of melanomas exist and may offer additional therapeutic options. The authors recommended that melanomas that originate on mucosal, acral, or chronic sun damage (CSD) skin should be investigated for KIT mutations and that imatinib is beneficial when tumors harbor KIT mutations, while it is ineffective when KIT is only amplified. Imatinib resistance may be mediated by NRAS mutations and increased KIT copy number [105]. Guo and Zhang presented a 58-year-old female diagnosed with stage III vaginal malignant melanoma. After local excision of the tumor and inguinal lymphadenectomy, the patient was treated with nivolumab. In six months of follow-up, neither regional recurrence nor distant metastases were identified [68]. In 2020, Anko et al. presented two patients with stage IB1 cervical and stage IV vaginal melanomas. Both patients were treated with nivolumab and showed a significant clinical response (the tumors nearly vanished) [104]. Tokita et al. documented the first and only case of nivolumab-treated primary amelanotic urethral melanoma. The diagnosis in the case report was primary malignant melanoma of the urethra with metastases to the left inguinal lymph nodes; stage 3 (cTxN1M0). The surgery was performed, and after finding new metastases and the failure of chemotherapy (DAV-Feron), the patient was treated with nivolumab for 10 cycles. The patient was free of disease in the next 20 months [70]. D'Angelo analyzed the largest dataset for anti-programmed death-1 therapy in GUMM and found that Nivolumab combined with ipilimumab appeared to have more efficacy than either agent alone, and while activity was lower in mucosal melanoma, the long-term safety was similar [63]. The reason for the aforementioned different responses in GUMM is unknown, although Furney et al. speculated that GUMM is less immunogenic due to a reduced mutational frequency [105]. Moya-Plana et al. discovered that treatment with immune checkpoint inhibitors showed positive results for GUMM, with ORR rates of about 10% for ipilimumab and 35% for pembrolizumab. This revealed similar therapeutic results as in cutaneous melanoma. Indeed, as previously described in cutaneous melanoma, Moya-Plana et al. demonstrated long-term anti-tumor effects in their cohort [64]. They found that anti-PD1 immunotherapy had a superior benefit-to-risk ratio than anti-CTLA-4 immunotherapy, recommending that anti-PD1 be used as the first treatment choice [64]. Indini et al. reported seven patients with metastatic melanoma of the female genital tract treated with immune-checkpoint inhibitors. In this trial, the response rate to immunotherapy was about 28%, with a mean (SD) survival of 6.1 (2.2) months. The authors concluded that the prognosis of this disease is poor. Despite the fact that the small sample size and short follow-up time prevented researchers from establishing any solid conclusions, they discovered that anti-PD-1 therapy is related to improved outcomes when compared to anti-CTLA4 therapy [65]. Sheng et al. emphasized that monotherapy with immune checkpoint agents has a low response rate in metastatic GUMM [66,67]. Mucosal melanoma is a highly vascular tumor, and previous clinical investigations found that the expression level of vascular endothelial growth factor (VEGF) was linked to poor outcomes in individuals with GUMM. VEGF has both immunosuppressive and angiogenic characteristics; however, Cui et al. demonstrated that antiangiogenic monotherapy had not shown substantial improvement compared with chemotherapy in melanoma [106].

Yan et al. assessed the therapeutic response of a first-line chemotherapy regimen (carboplatin + paclitaxel) combined with an antiangiogenic medication (bevacizumab) in patients with GUMM in the first randomized control trial. When compared to chemotherapy alone, adding bevacizumab to carboplatin with paclitaxel improved progression-free survival from 3.0 to 4.8 months and overall survival from 9.0 to 13.6 months [67].

In 2021, Shoushtari proposed a four-arm clinical trial comparing chemotherapy with bevacizumab vs. PD-1 blockade, with or without CTLA-4 or vascular endothelial growth factor receptor (VEGFR) inhibitor, based on past research and trials [107]. According to Shoushtari’s explanation, the necessity for such research stems from the fact that the last large randomized clinical trial was conducted in China, and the results may not be relevant to a more heterogeneous population [66,67]. He also mentioned that it is still unclear if combining bevacizumab with platinum chemotherapy would benefit the patients who have already advanced on checkpoint inhibitor treatment. He also argues that the findings of such a study might affect the treatment approach of a subset of patients, such as those who do not have access to PD-1-based medicines, patients with autoimmune illnesses, and those using immunosuppressive drugs. In these groups, the immediate impacts of the proposed trial mentioned above on treatment would be significant if carboplatin, paclitaxel, and bevacizumab could have been chosen as a frontline therapy regimen in the absence of any other contraindications [108]. Compared to observation alone, previous studies showed that temozolomide-based chemotherapy and high dosage IFN-a2b (HDI) are efficacious and safe as adjuvant treatments for resected GUMM. However, Lian et al. claimed that in patients with excised mucosal melanoma, HDI is less efficacious than temozolomide-based treatment in terms of relapse-free survival (RFS). For individuals with resected GUMM, the temozolomide plus cisplatin regimen may be a superior option [109].

## Conclusions

A comprehensive review of the available literature reveals a lack of standard protocol in approach to diagnosis and treatment of GUMM. Most importantly, there is a lack of a standard stage scheme and pathological reporting for mucosal MM that needs to be addressed. It is not clear how MM develops in mucosal tracts; however, migration theories have merit. Similarities in genetic signatures of mucosal and skin MM suggest similar mechanism of development; however, unlike skin melanoma, there is less BRAF mutation and more of PI3K/AKT/mTOR pathway alterations in mucosal MM. Studies suggest that prolonged administration of chemotherapy (i.e., methotrexate) and immune-modulating agents (i.e., natalizumab) may be a risk factor for GUMM. 

Based on our review, mucosal (including GU) malignant melanomas are aggressive diseases that show variable responses to conventional cutaneous melanoma regimens. In light of absence of definite adjuvant therapy, the stage at the time of diagnosis of mucosal MM and proper surgical removal has a central role in the prognosis and survival of patients. Observation of higher rates of KIT alteration suggests a higher response rate to targeted KIT therapy; unfortunately, such response may be self-limited. Immune checkpoint therapy also appears promising; however, the response rate appears to be lower than cutaneous melanomas.
